# Experimental Study on Shear Capacity of High Strength Reinforcement Concrete Deep Beams with Small Shear Span–Depth Ratio

**DOI:** 10.3390/ma13051218

**Published:** 2020-03-09

**Authors:** Jun-Hong Zhang, Shu-Shan Li, Wei Xie, Yang-Dong Guo

**Affiliations:** School of Civil Engineering and Communication, North China University of Water Resources and Electric Power, Zhengzhou 450046, China; zhangjunhong@ncwu.edu.cn (J.-H.Z.); xwei@ncwu.edu.cn (W.X.); gyangdong888@163.com (Y.-D.G.)

**Keywords:** high-strength reinforcement concrete, deep beam, cracking load, shear capacity, strut-and-tie model

## Abstract

This study aimed to investigate the shear capacity performance for eight deep beams with HTRB600 reinforced high strength concrete under concentrated load to enable a better understanding of the effects of shear span–depth ratio, longitudinal reinforcement ratio, vertical stirrup ratio and in order to improve design procedures. The dimension of eight test specimens is 1600 mm × 200 mm × 600 mm. The effective span to height ratio *l_0_*/*h* is 2.0, the shear span–depth ratio *λ* is 0.3, 0.6 and 0.9, respectively. In addition, the longitudinal reinforcement ratio *ρ*_s_ is set to 0.67%, 1.05%, 1.27%, and the vertical stirrup ratio is taken to be 0%, 0.25%, 0.33%, 0.5%. Through measuring the strain of steel bar, the strain of concrete and the deflection of mid-span, the characteristics of the full process of shear capacity, the failure mode and the load deflection deformation curve were examined. The test results showed that the failure mode of deep beams with small shear span–depth ratio is diagonal compression failure, which is influenced by the layout and quantity of web reinforcement. The diagonal compression failure could be classified into two forms: crushing-strut and diagonal splitting. With decreasing of shear span–depth ratio and increasing longitudinal reinforcement ratio, the shear capacity of deep beams increases obviously, while the influence of vertical web reinforcement ratio on shear capacity is negligible. Finally, the shear capacity of eight deep beams based on GB 50010-2010 is calculated and compared with the calculation results of ACI 318-14, EN 1992-1-1:2004 and CSA A23.3-04, which are based on strut-and-tie model. The obtained results in this paper show a very good agreement with GB50010-2010 and ACI 318-14, while the results of EN 1992-1-1:2004 and CSA A23.3-04 are approved to be conservative.

## 1. Introduction

According to GB 50010-2010 [1], beams can be known as deep beams when the effective span to height ratio *l_0_*/*h* ≤ 2 or continuous beams *l_0_*/*h* ≤ 2.5. The difference between the deep beam and the general beam is that the shear force is mainly transmitted by the way of arch action after the diagonal crack occurs. Since the 1980s, the special research group of deep beams in People’s Republic of China (P.R.C) has carried out numerous experimental researches on simply supported deep beams, continuous deep beams [2] and deep beams with web openings [3]. On this basis, a practical and unified calculation formula of shear capacity which was suited to reinforced concrete deep beams, short beams and shallow beams was put forward [4,5]. Since the strut-and-tie Model (STM) came forward in 1985s [6], STM have been generally recognized as a good design tool to meet the structural stress characteristics [7,8]. Based on STM, many scholars come up with theoretical methods for calculating the shear capacity of deep beams. For instance, compression field theory (CFT) and modified compression field theory (MCFT) [9,10], the theory of softened strut and tie model (SSTM) considering the compression softening of concrete [11,12] and strut and tie model based on the crack band theory [13,14], and so on. These promoted the development and application of STM. At present, most current design codes, such as ACI 318-14 [15], EN 1992-1-1:2004 [16], CSA A23.3-04 [17] have recommended the STM approach as a design tool for deep beams. In addition, Tan et al. proposed the Tan–Tang model [18] and Tan–Cheng model [19], considering the influence of size effect on the shear capacity of deep beams, and the prediction results of the proposed calculation formula are in good agreement with the test values.

In recent years, many experiments and numerical analysis have been carried out the influence of different design parameters on the shear capacity of deep beams. In reference [20,21,22,23], based on the research of the failure mode of deep beams, the influence of concrete strength, shear span–depth ratio, reinforcement ratio, loading plate and other factors on the shear capacity of deep beams has been conducted. The influence of high strength concrete on the shear capacity of deep beams has been discussed in references [24,25]. Moreover, in reference [26,27], through the analysis of nonlinear finite element numerical, the stress characteristics and failure mechanism of deep beam structure are further obtained, which provides theoretical support for the design of deep beam. Considering the fracture and damage characteristics of concrete as a typical brittle material [28,29,30], some scholars studied deep beams by changing the composite structure of deep beams or adding new materials. In reference [31], the spatial steel frame is set in the deep beams and the authors got the research conclusion that the shear capacity and deformation capacity of deep beams with spatial steel frame are greatly improved when compared with general beams. In 2019, Wu et al. [32] completed fifteen shear tests of lightweight aggregate reinforced concrete deep beams. In the meantime, the failure process of members and the applicability of relevant calculation models were analyzed. Gao et al. [33,34] discussed the influence rule of steel fiber content on the shear behavior of deep beams. On top of this, the calculation formulas of normal section crack resistance and diagonal section crack resistance of steel fiber reinforced concrete (SFRC) deep beams have been derived. In 2019, Ma et al. [35] through the test of four hybrid fiber reinforced concrete (HFRC) deep beams and one reinforced concrete (RC) deep beam got the conclusion that under the same web reinforcement ratio, the ultimate load and deformation of the HFRC deep beams were better than those of the RC deep beam.

Numerous research studies on shear behavior of reinforced concrete members have been conducted. However, there is still discord regarding the transfer mechanisms and influencing parameters. In this paper, Eight C50 concrete deep beams with HTRB600 high-strength reinforcement are tested and studied. The effects of shear span–depth ratio, longitudinal reinforcement ratio and vertical stirrup ratio on the failure mode, shear capacity and mid-span deflection of deep beams are analyzed. Finally, GB 50010-2010, ACI 318-14, EN 1992-1-1:2004 and CSA A23.3-04 are used to derive the shear capacity of deep beams and study the applicability of each code’s equation.

## 2. Test Overview

### 2.1. Experiment Material

In this test, C50 commercial concrete is used to pour the deep beam components, six 150 mm × 150 mm × 150 mm concrete blocks are poured at the same time to test the compressive strength and splitting tensile strength, and six 150 mm × 150 mm × 300 mm concrete blocks are poured to examine the compressive strength and compressive modulus of elasticity of prism. HTRB600 grade steel bars are used as the bottom longitudinal bars, HRB400E grade steel bars are used as the vertical stirrups and horizontal distributing reinforcements. The mechanical properties of concrete and reinforcement are shown in Table 1 and Table 2.

### 2.2. Specimen Design

**Figure 1:**
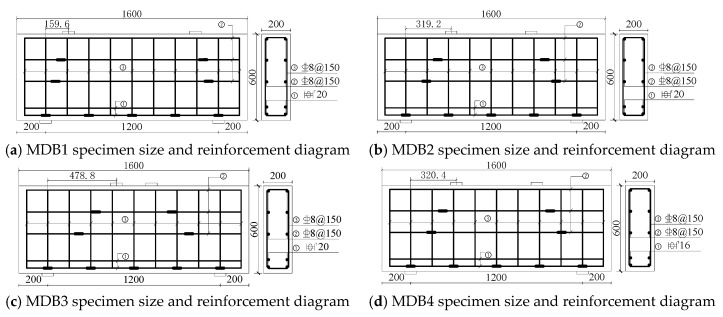

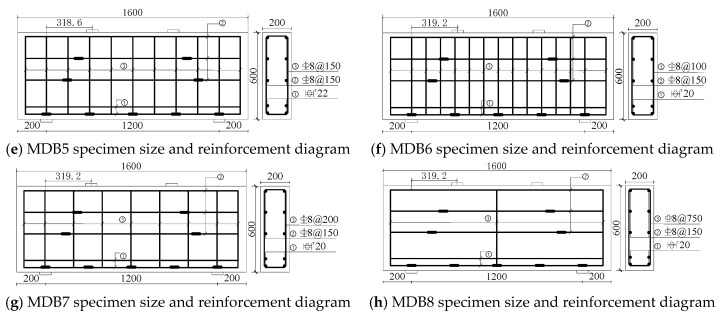
Geometric dimension of test specimen and layout of steel strain gauge.

Eight deep beam members (MDB1-MDB8) have been cast in the experiment. The dimension of the test specimen *b* × *h* × *l* is 200 mm × 600 mm × 1600 mm, the geometric dimension and reinforcement layout are shown in Figure 1. The design parameters include shear span–depth ratio *λ*, longitudinal reinforcement ratio *ρ*_s_ and vertical stirrup ratio *ρ*_sv_, the effective span to height ratio *l*_0_/*h* takes the value of 2 in this study. See Table 3 for test specimen parameters.

### 2.3. Test Setup and Instrumentation

**Figure 2:**
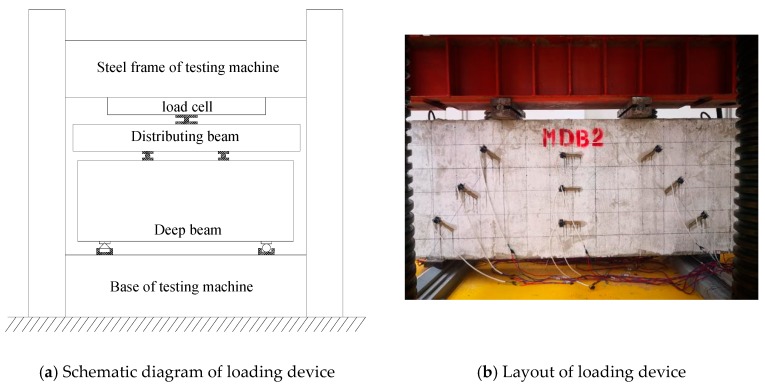
Test loading device.

YJW-10000 hydraulic compression testing machine is used, with load capacity of 1000 tons. The test loading device is shown in Figure 2. The size of the steel packing plates is 80 mm × 200 mm × 20 mm. The vertical load is transferred to the test component through the distribution beam and the steel packer plates. First, preload the test specimen to ensure the normal operation of measuring instruments. The formal loading level is divided into 15–20 levels with each level being 100 kN, and the loading increment rate is 1 kN/s. The load value of each level is measured and recorded after each level passes 3 min steady period. In order to determine the cracking load precisely, the loading speed should be reduced before the initial diagonal crack is predicted [36,37]. When the loading is reaching the ultimate load, then a continuous and slow loading will be commenced until the destruction of specimen.

**Figure 3:**
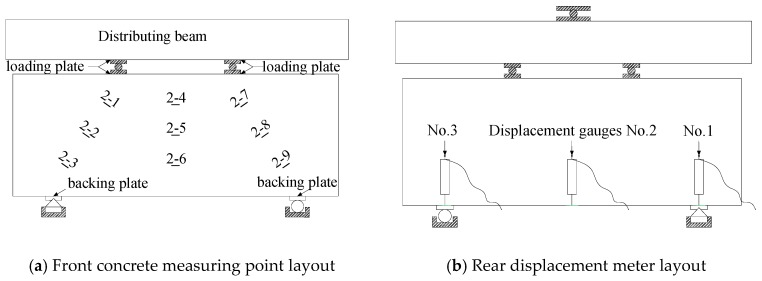
MDB2 rebar and concrete strain gauge layout.

Nine concrete strain gauges are placed on the surface of the deep beam specimen, of which three are placed on the mid-span normal section and three are positioned on the left and right diagonal sections respectively, which are used to keep track of the concrete strain of the mid-span normal section and the shear span diagonal section of the deep beam. The strain gauges are used for the longitudinal reinforcements and horizontal distributing reinforcements of the deep beam specimens to record the strain variation of the steel bars. Three displacement meters are placed at both ends of the mid-span and support of the test specimen to monitor the mid-span deflection. The locations of strain gauge and displacement gauge measuring points are shown in Figure 3. The data of concrete strain, reinforcement strain and displacement are collected via the Isolated Measurement Pods (IMP) data automatic acquisition system; the concrete crack width is measured by using KON-FK (N) crack observation instrument (Beijing Koncrete Engineering Testing Technology CO., Ltd, Beijing, China) with a minimum scale of 0.01 mm, and the crack’s propagation status is also recorded at the same time.

## 3. Behavior of Test Specimens and Failure Modes

### 3.1. Failure Modes

**Figure 4:**
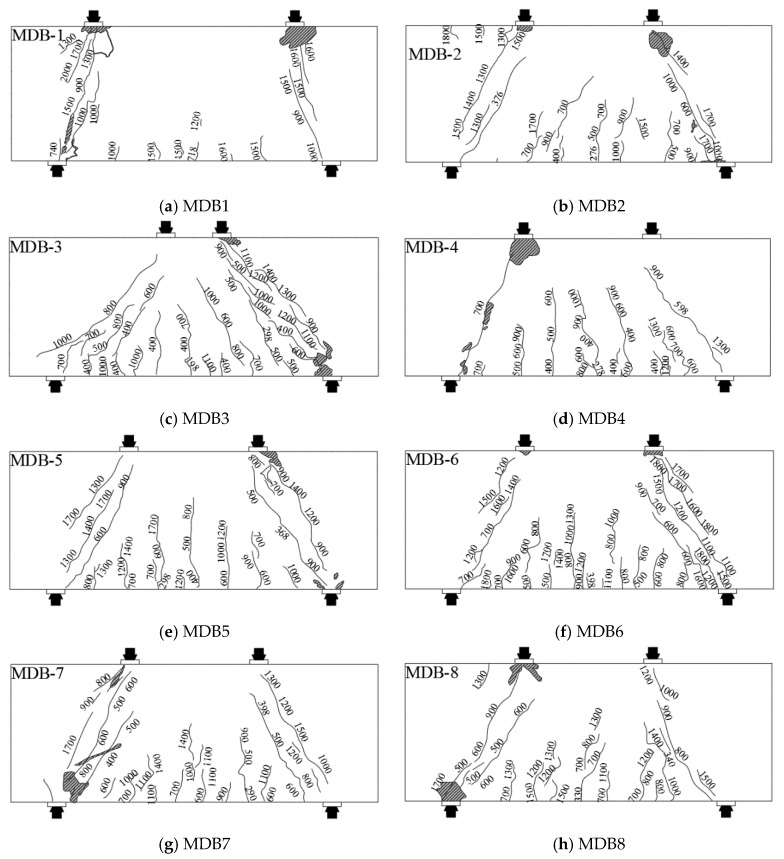
Crack patterns at failure.

Although the parameters of deep beam specimens are different, they have experienced three stages from the initial loading to the final failure: cracking, critical cracking and ultimate limit. The characteristic load of each stage and final failure mode of the specimen are shown in Table 4. The normal section cracking load of the high-strength reinforced concrete deep beam is approximately 10–20% of the ultimate load, while the diagonal section cracking load is about 20–40% of the ultimate load. The failure mode of deep beams with small shear span–depth ratio is diagonal compression failure. Due to the configuration and amount of web reinforcement, the diagonal compression failure mode of MDB1–7 specimen is crushing-strut, while that of MDB8 specimen is diagonal-splitting [38]. The crack patterns of the specimen when they are finally reach destruction are presented in Figure 4.

### 3.2. Failure Process of Test Specimens

We take specimen MDB3 as an example to illustrate the failure process of deep beams with small shear span–depth ratio. From the beginning of loading to the first crack, the specimen is in the elastic deformation stage. When the applied load reaches 12.61% of the ultimate load, the vertical flexural crack start appearing near the mid-span of the specimen. The crack length is about 1/6 of the beam height and the crack width is about 0.04 mm. The cracking load of the normal section is recorded to be 99 kN. More than ten new flexural cracks gradually formed around the mid-span as the loading increases, and the crack widths increase slowly, but these cracks gradually spread in the longitudinal direction until failure. Some flexural cracks extended to 2/3 or 1/2 of the beam height, and the maximum crack width was measured 0.28 mm.

When the imposed load increases 18.98% of the ultimate load, the first diagonal crack of 0.06 mm width with about 1/3 of the beam height is observed between the right loading plate and the bearing plate, and the diagonal section crack load is 149 kN. When the load continues to increase to 25.47% of the ultimate load, a new diagonal crack developed at about 1/3 of the beam height on the left support side along the line between the loading plate and the bearing plate. At the same time, a new diagonal crack appears at the right support side along the line between the loading plate and the bearing plate, which is parallel to the original diagonal crack, and this crack rapidly extends from the bottom of the beam to 2/3 of the beam height, with a crack width of about 0.12 mm. Since then, with the increase of the load, the diagonal cracks on both sides spread in the diagonal direction, and the width of the cracks also increases. When the load reaches 57.32% of the ultimate load, a full-length diagonal crack with crack width of 0.22 mm is observed on the left support side. A few new diagonal cracks on the inner side of the line between the right loading plate and the bearing plate turn up, with a maximum crack width of 0.82 mm. When the load finally reaches 95.54% of the ultimate load, the width of the main diagonal crack on the left support side is about 0.62 mm, and multiple diagonal cracks are formed from the web to the top of the beam on the right support side, with the maximum crack width being 1.66 mm. There is a slight concrete splitting sound from the specimen with the increase of the applied load. When the load reaches the ultimate load, the beam failed suddenly and violently as the concrete in the compression zone crushed.

**Figure 5:**
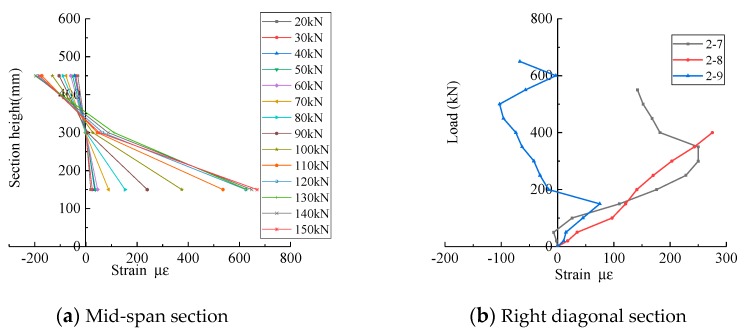
Surface concrete strain of test specimen MDB3.

**Figure 6:**
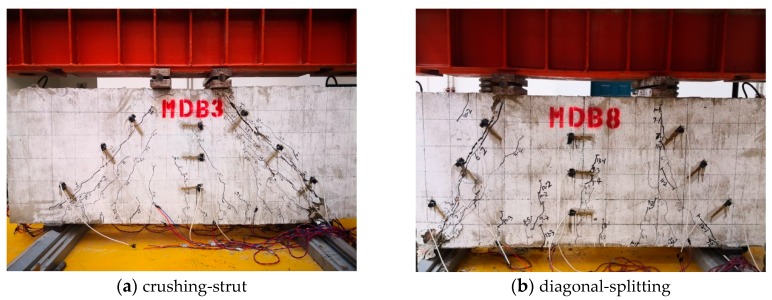
Failure mode of deep beam.

The test results show that the concrete strain of the normal section does not meet the plane section assumption at the mid-span of the specimen [8,23,39], at the same time, with the development of cracks, the neutral axis moves up gradually, and a bottle-shaped graph is formed for the concrete strain of the inclined section. Taking MDB3 as an example, Figure 5a presents the variation of mid span concrete strain of the normal section against beam height in the elastic stage, and the influence of applied concentrated load on the right side concrete strain of the diagonal section is depicted in Figure 5b. It is obvious that the appearance of diagonal cracks results in a stress redistribution in the specimen, and forms a tension arch force system with the consideration of the concrete between the loading point and the supports as the soffit. With increasing load, the compression load of the beam soffit and apex of the arch also increases. As a result, there are plenty of diagonal cracks around the beam web area, which are roughly parallel to the line between the bearing point and the loading point. The concrete peels off from the top to the bottom. In the end, the soffit of concrete beam is crushed, and the final form of failure is crushing-strut. The failure mode of MDB3 is shown in Figure 6a.

No vertical stirrups have been placed for the specimen MDB8, and the quantity of web reinforcement is considerably lower than the other specimen’s. With the formation of tension arch force system and increase of the imposed load, a full-length split crack which is roughly coincident with the main diagonal crack at the beam right hand side suddenly comes out, and the final failure manner is diagonal-splitting. The failure mode of MDB8 is displayed in Figure 6b.

### 3.3. Influencing Factor Analysis

#### 3.3.1. Shear Span–Depth Ratio

**Figure 7:**
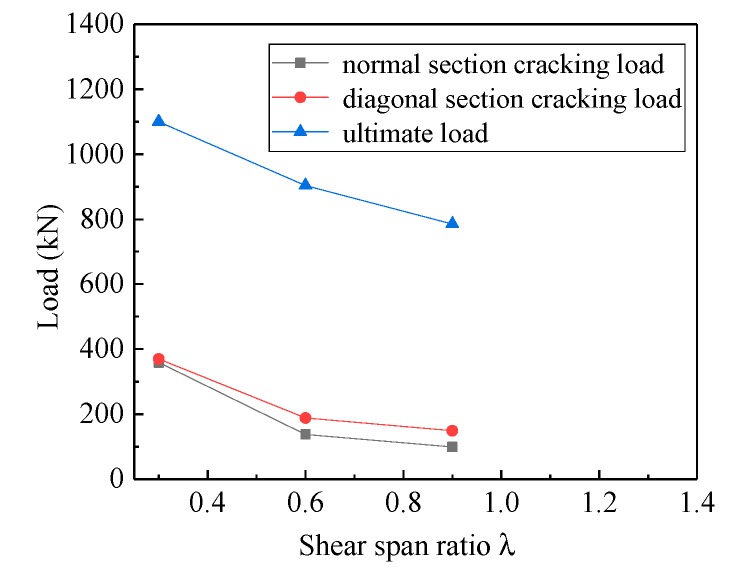
Relation curve between λ and load.

The shear span–depth ratio is a critical in controlling the shear capacity behavior of deep beams [40]. Through the study of three deep beam specimens, MDB1 (λ = 0.3), MDB2 (λ = 0.6), and MDB3 (λ = 0.9), the influence of shear span–depth ratio on the mechanical performance of high-strength reinforced concrete deep beams was analyzed. The relationship between normal section cracking load, diagonal section cracking load, ultimate load and shear span–depth ratio is illustrated in Figure 7. The experimental results confirms that, the normal section cracking load, diagonal section cracking load and ultimate load of the specimen all increase as the shear span–depth ratio declines, which is consistent with the test conclusion in literature [23,25,32].

Compared with MDB1, the cracking load of MDB2 and MDB3 reduced by 61.55% and 72.41% respectively, the diagonal load decreased by 49.19% and 59.73% respectively, and the ultimate load went down by 17.86% and 28.63% respectively. This decrease in the ultimate load can be accounted for by the fact that the tied-arch action becomes more ineffective due to the decreased angle between the diagonal concrete strut and the longitudinal axis (changes the width of the strut) when the shear span–depth ratio increases. With the increase of the distance between loading point and bearing point, the effectiveness of the arch action was reduced.

**Figure 8:**
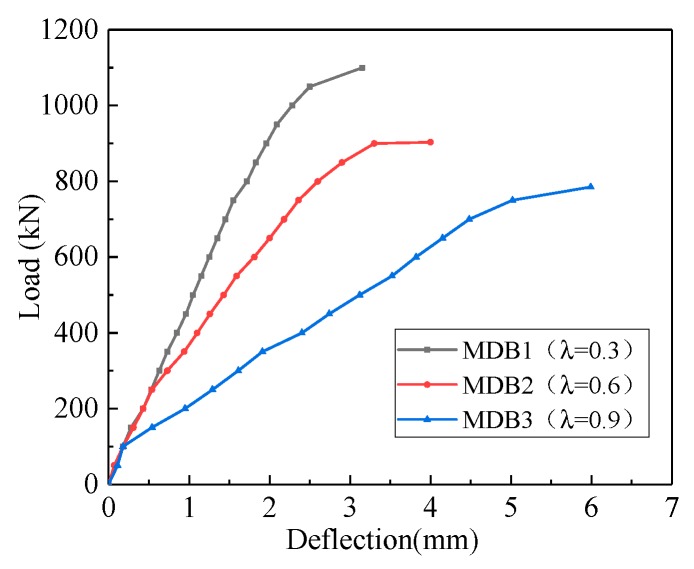
Relation curve between λ and deflection.

The influence of shear span–depth ratio on the mid-span deflection is shown in Figure 8. As expected, the initial stiffness and overall response of the specimens differ depending on the shear span–depth ratio. As the shear span–depth ratio increases, the mid-span deflection of deep beam is expected to increase. The maximum deflection of MDB1, MDB2 and MDB3 is 3.15 mm, 4.0 mm and 5.99 mm respectively. With the increase of shear span–depth ratio, the bending deformation of test specimens is visual obviously, the effectiveness of arch action is dropped, and the amount of cracks within the shear span area is increased, which leads to the dramatically decrease of the stiffness degradation.

**Figure 9:**
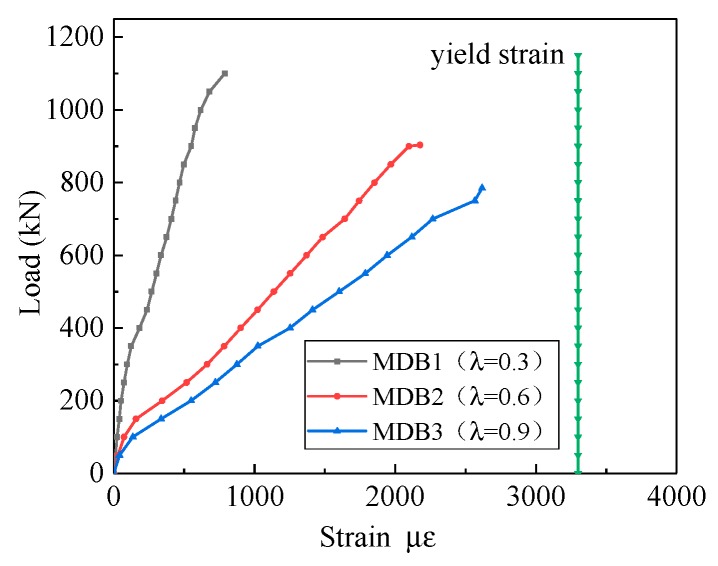
Relation curve between λ and longitudinal reinforcement strain.

The relationship between the strain of the longitudinal reinforcement and the shear span–depth ratio is presented in Figure 9. Prior to the specimen cracking, the shear force is mainly borne by the concrete, and the stress of the reinforcement is very small. Under the same level of load, the strain of longitudinal reinforcement increases with increasing shear span–depth ratio. Under the ultimate load, the longitudinal bars do not reach its yield stress [41].

**Figure 10:**
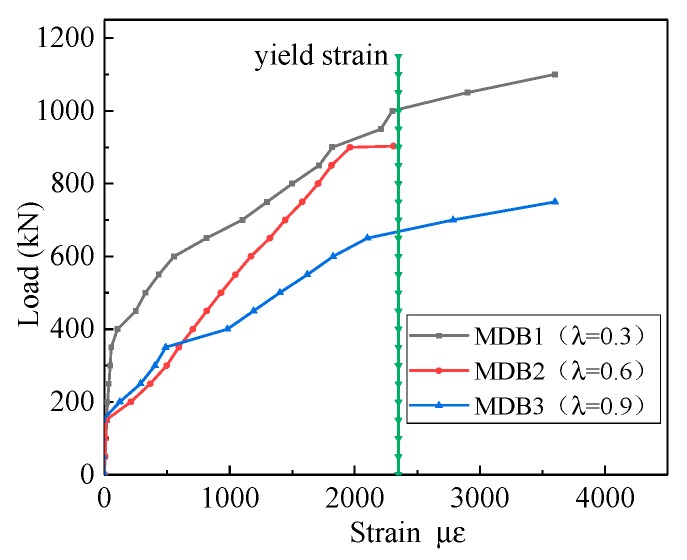
Relation curve between λ and horizontal distributing reinforcement strain.

Figure 10 displays the variation of the horizontal distributing reinforcement stain against the shear span–depth ratio. The stress of horizontal distributing reinforcement is very small before the diagonal section cracks. The shear force is mainly carried by the concrete, but since the diagonal cracks occurred, the horizontal distributing reinforcements gradually become involved to take a portion of shear load together with the concrete. With the increase of the applied loading, the strain of the horizontal distributing reinforcement through the diagonal cracks increases rapidly, and finally achieves the yield stress. The role of the horizontal distributing reinforcement is fully employed. The strain of horizontal distributing reinforcement increases with increasing shear span depth ratio.

**Figure 11:**
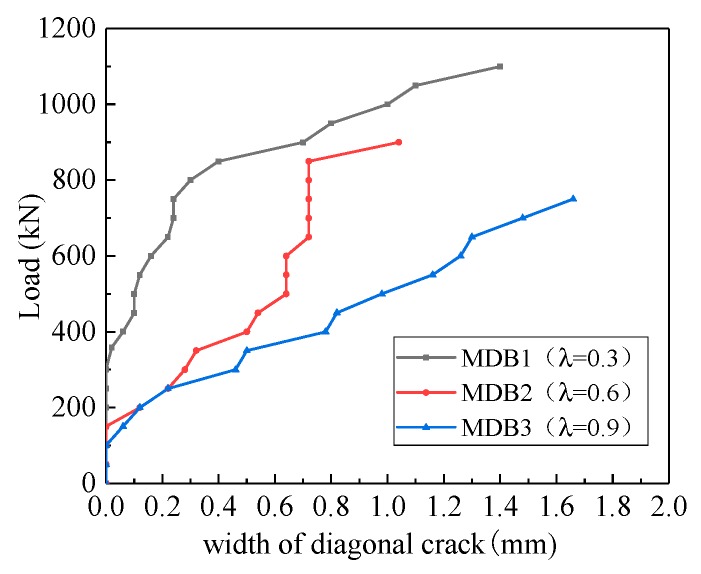
Relation curve between λ and maximum crack width.

Figure 11 exhibits that the variation of the diagonal crack width versus the shear span–depth ratio. At the initial stage of loading, flexural cracks and diagonal cracks appear successively in the mid-span and the flexural shear section. The width of flexural cracks is small, and the crack will stop spreading in the vertical direction after slowly extending to 1/2–2/3 of the total height of beam. Once the diagonal crack happens, it will expand quickly, and the crack width is much larger than that of the flexural crack. If a certain amount of web reinforcement is provided, there are numerous diagonal cracks, and the beam is crushed to failure in the compression area. With the increase of shear span–depth ratio, the maximum crack width corresponding to the same loading also increases. The maximum crack width of MDB1–MDB3 is 1.40 mm, 1.04 mm and 1.66 mm respectively.

#### 3.3.2. Longitudinal Reinforcement Ratio

**Figure 12:**
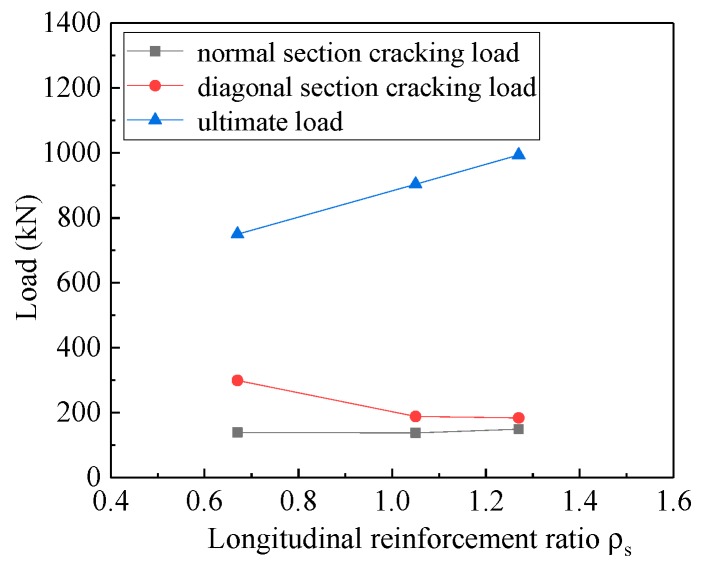
Relation curve between *ρ*_s_ and load.

Based on the experimental study of MDB4 (*ρ*_s_ = 0.67%), MDB2 (*ρ*_s_ = 1.05%), MDB5 (*ρ*_s_ = 1.27%), the influence of longitudinal reinforcement ratio on the mechanics performance of high-strength reinforced concrete deep beams is analyzed. The curve of the relationship between the normal section cracking load, the diagonal section cracking load, the ultimate load and the longitudinal reinforcement ratio of the test specimen is shown in Figure 12. With the increase of the longitudinal reinforcement ratio from 0.67% to 1.27%, the influence on the normal section cracking load and the diagonal section cracking load of the test specimen is negligible, while the ultimate load increases significantly, which agrees well with the findings of the previous study [24]. Compared with MDB4, the cracking load of MDB2 is nearly the same, the cracking load of MDB5 is increased by 7.19%, while the cracking load of diagonal section is decreased by 37.12% and 38.46%, and the ultimate load is increased by 20.46% and 32.49%, respectively. The longitudinal tension reinforcement at the bottom of beam affects the shear capacity of the beam via the pin bolt action, which not only restrains the development of the inclined crack, but also improves the shear transfer performance between the inclined crack interfaces. With the increase of the longitudinal reinforcement ratio, ultimate load increases significantly.

**Figure 13:**
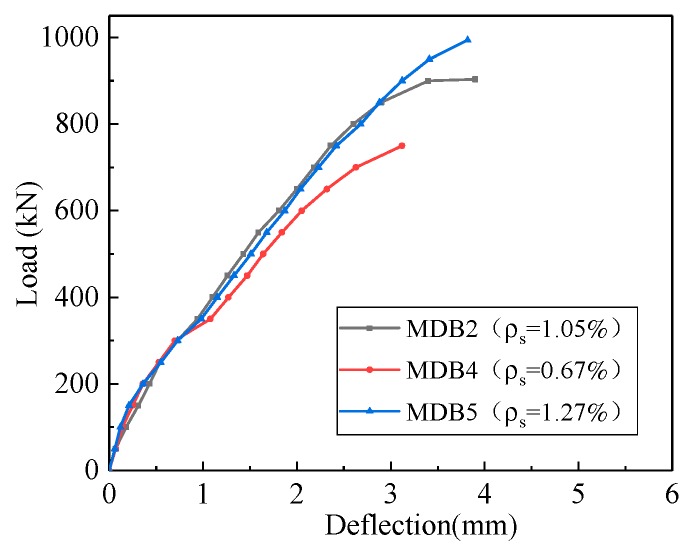
Relation curve between *ρ*_s_ and deflection.

Figure 13 depicts the relationship between the deflection and longitudinal reinforcement ratio. The mid-span deflection corresponding to the equal load has no variations with the increase of longitudinal reinforcement ratio. The maximum mid-span deflection of MDB4, MDB2 and MDB5 is 3.12 mm, 4.0 mm and 3.82 mm respectively. In shallow beams, the longitudinal reinforcement ratio has a significant impact on the stiffness of the beam; while in deep beams, the shear failure likely occurs, and the longitudinal reinforcement ratio has little influence on the stiffness of the beam. Therefore, the variation of the longitudinal reinforcement ratio has less impact on the deflection of the deep beam.

**Figure 14:**
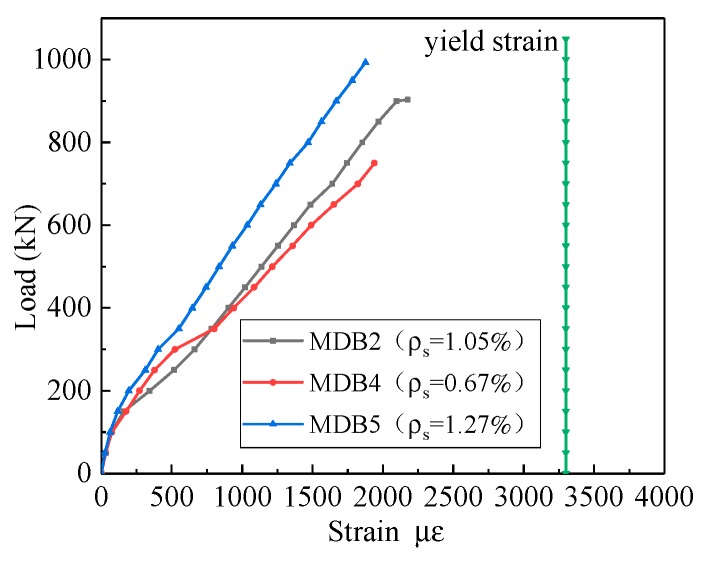
Relation curve between the *ρ*_s_ and longitudinal reinforcement strain.

The relationship between the strain of the longitudinal reinforcement and the longitudinal reinforcement ratio is displayed in Figure 14. Before the specimen cracks, the shear force is mainly supported by the concrete, and the stress of the reinforcement is very small. This observation reveals that reinforcement had a minimal influence on the occurrence of diagonal cracks.

It shows clearly that higher longitudinal reinforcement ratio yields the smaller strain of the longitudinal reinforce at the same loading. Under the ultimate load, the yield strain of the longitudinal bars does not happen.

**Figure 15:**
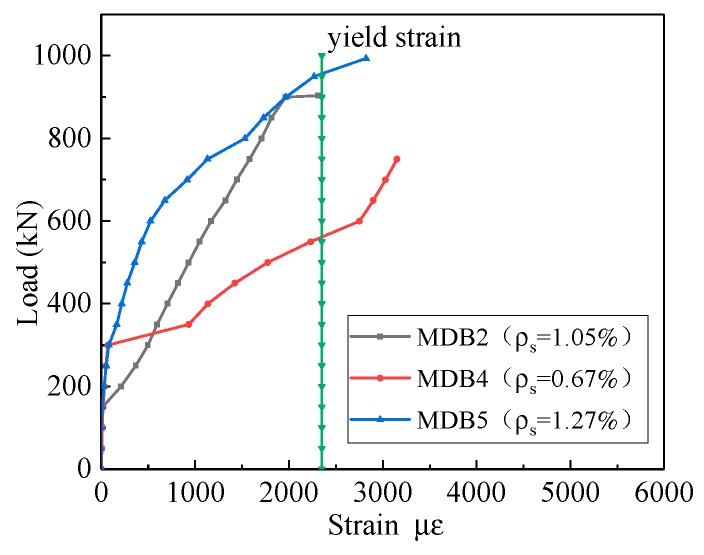
Relation curve between *ρ*_s_ and horizontal distributing reinforcement strain.

The curve of the relationship between the horizontal distributing reinforcement strain and the longitudinal reinforcement ratio is presented in Figure 15. Since the diagonal section cracks, the stress of the horizontal distributing reinforcement rapidly increases and passes the yield point which is due to the full utilization of the horizontal distributing reinforcement. The strain of the horizontal distributing reinforcement corresponding to the equal load declines with the increase of the longitudinal reinforcement ratio.

**Figure 16:**
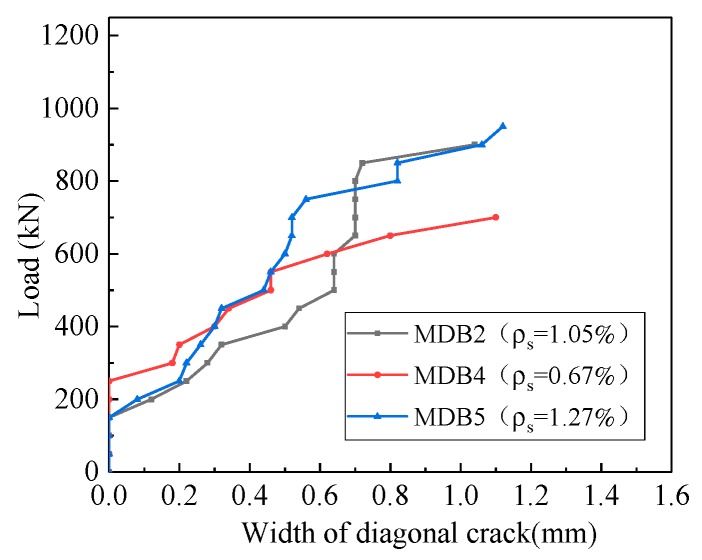
Relation curve between *ρ*_s_ and maximum crack width.

The curve of the relationship between the diagonal crack width and the longitudinal reinforcement ratio is presented in Figure 16. At the initial stage of loading, flexural cracks and diagonal cracks appear successively in the mid-span and the flexural shear section. The width of flexural cracks is small, and it will not continue to spread after tardily extending to 1/2–2/3 of the beam height along vertical direction. MDB4, MDB2 and MDB5 specimens have multiple diagonal cracks parallel to the link line between the bearing point and the loading point. Finally, the compression strut is destroyed by crushing. With the increase of longitudinal reinforcement ratio, the maximum crack width corresponding to the same loading is basically same. The maximum crack width of MDB4, MDB2 and MDB5 is 1.1 mm, 1.04 mm and 1.12 mm respectively.

#### 3.3.3. Vertical Stirrup Ratio

**Figure 17:**
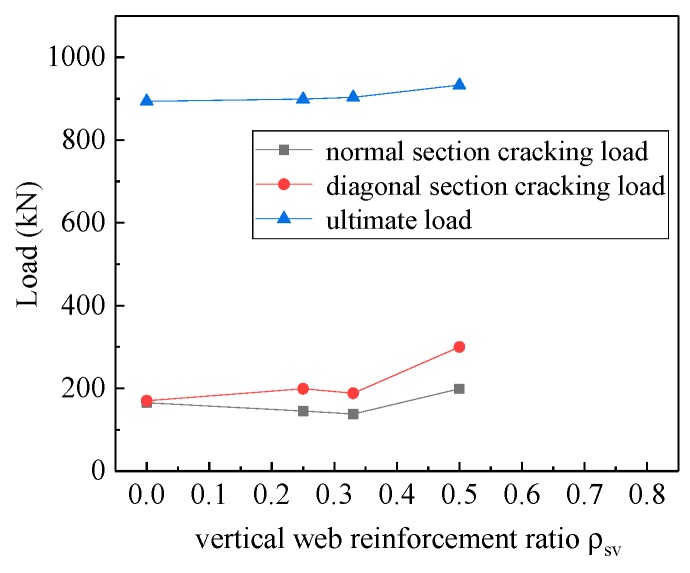
Relation curve between *ρ*_sv_ and load.

The test through the study of four deep beam members MDB6 (*ρ*_sv_ = 0.50%), MDB2 (*ρ*_sv_ = 0.33%), MDB7(*ρ*_sv_ = 0.25%), MDB8 (*ρ*_sv_ = 0) to explore the vertical distribution effect of reinforcement on the mechanical performance of high-strength reinforced concrete deep beams with small shear span–depth ratio. The curve of the relationship between the normal section cracking load, the diagonal section cracking load, the ultimate load and the vertical stirrup ratio of the test pieces are shown in Figure 17. With the increase of the vertical stirrup ratio from 0% to 0.50%, the normal section cracking load, the diagonal section cracking load and the ultimate load of the deep beam slightly change, indicating that the vertical stirrup ratio does not provide significant influence on the deep beam shear capacity because in deep beams most of the applied shear force is transferred by the strut-and-tie action. Compared with the test specimen MDB8, the normal cross-section cracking load of the test specimens MDB7 and MDB2 decreases by 12.12% and 16.36% respectively; while the normal cross-section cracking load of MDB6 increases by 20.60%. When compared with MDB8, the crack load of the diagonal sections of the test specimens MDB7, MDB2 and MDB6 increases by 17.05%, 10.58% and 76.47%, respectively. The ultimate loads of test specimens MDB7, MDB2 and MDB6 increase by 0.61%, 1.12% and 4.42%, respectively. Vertical stirrups can control the development and width of diagonal cracks, so as to improve the shear strength and ductility of deep beams. However, these effects are negligible [1] because in deep beams most of the applied shear force is transferred by the strut-and-tie action.

**Figure 18:**
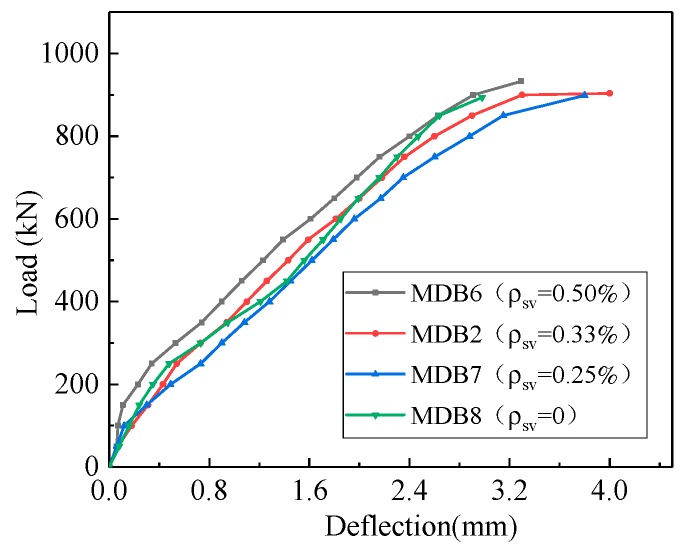
Relation curve between *ρ*_sv_ and deflection.

The relation curve between deflection and vertical stirrup ratio is shown in Figure 18. The mid-span deflection corresponding to the same loading basically remains unchanged with the increase of vertical stirrup ratio. The maximum deflection of MDB6, MDB2, MDB7, and MDB8 mid-span are 3.29 mm, 4.0 mm, 3.8 mm and 2.98 mm, respectively.

**Figure 19:**
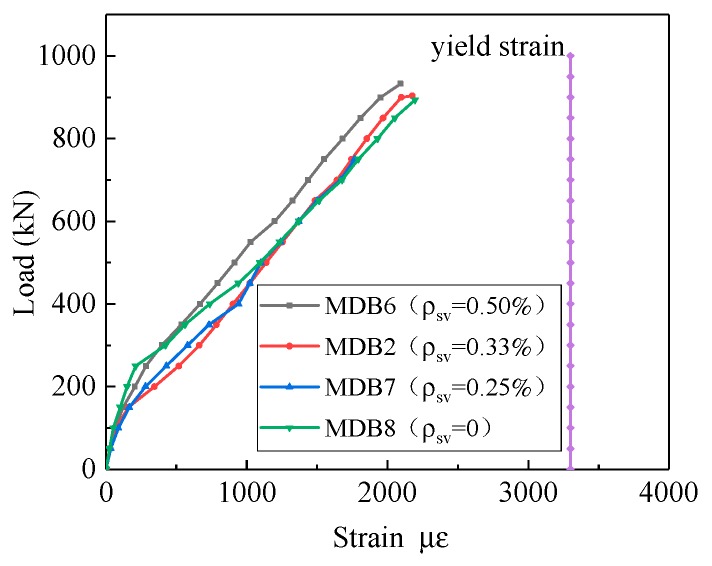
Relation curve between *ρ*_sv_ and longitudinal reinforcement strain.

The relation curve between the strain of the longitudinal reinforcement and the vertical stirrup ratio is illustrated in Figure 19. Prior to the specimen cracking, concrete resists predominantly the applied shear stress and shear reinforcement carries almost zero stress. But further increase of vertical stirrup ratio does not enhance the strain of the longitudinal reinforcement with a constant loading, and all the longitudinal bars have not yielded.

**Figure 20:**
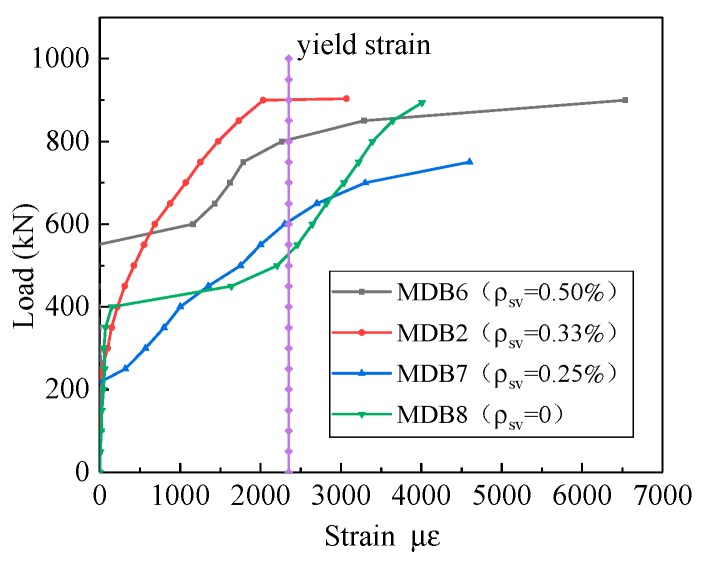
Relation curve between *ρ*_sv_ and horizontal distributing reinforcement strain.

The relationship between the strain of horizontal distributing reinforcement and the vertical stirrup is shown in Figure 20. After the inclined section cracks, the stress of horizontal distribution bar rapidly increases and finally yields, that is, the role of horizontal distributing reinforcement is fully played. With the increase of vertical stirrup ratio, the strain of horizontal distributing reinforcement corresponding to the same loading also increases.

**Figure 21:**
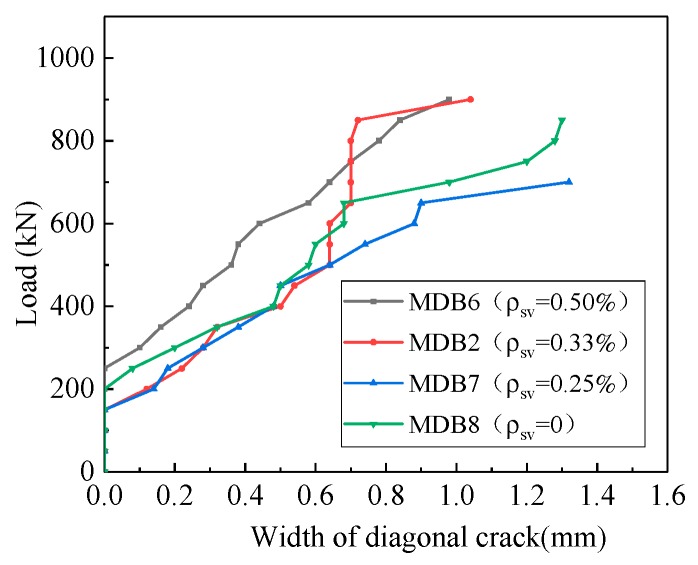
Relation curve between *ρ*_sv_ and maximum crack width.

Figure 21 shows the relationship between diagonal crack width and vertical stirrup ratio. At the initial stage of loading, flexural cracks and diagonal cracks successively turn up at the mid-span and flexural shear section. The width of flexural cracks is relatively small, and it will not continue to expand after slowly propagated to 1/2–2/3 of the beam height along the vertical direction. MDB6, MDB 2 and MDB 7 test specimens have multiple diagonal cracks parallel to the link line between the bearing point and the loading point. Finally, strut compression failure occurred due to crushing of the concrete between inclined cracks; the MDB8 specimen gradually formed the largest diagonal crack width, and finally was destroyed along the full length diagonal splitting. It is seen that the increase of the vertical stirrup ratio, always leads to corresponding decreases in the maximum crack. The maximum crack width of MDB 6, MDB 2, MDB 7 and MDB 8 were 0.98 mm, 1.04 mm, 1.32 mm and 1.3 mm respectively.

## 4. Design Method of Shear Capacity

The design method of the deep beam in the Chinese Code (GB50010-2010) is a semi-empirical and semi-theoretical calculation formula obtained on the basis of a large number of experiments. The shear span–depth ratios are all taken as 0.25, without considering the role of the stirrups.

**Figure 22:**
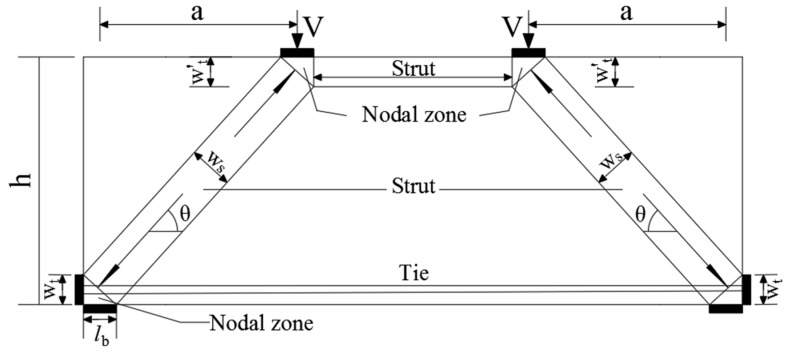
Simplified calculation of strut-and-tie model.

For the derivation of shear force of deep beams with small shear span–depth ratios, Canadian and European concrete structures design codes have adopted strut-and-tie model into the analysis and design of concrete members. The corresponding design method is given in Appendix A of American Code ACI318-14. The design process reasonably takes into account the factors of concrete strength, shear span–depth ratio, web reinforcement ratio, and loading conditions. The strut-and-tie mechanism and the size of each element are shown in Figure 22.

### 4.1. China’s GB50010-2010 Code

GB50010-2010 proposes a semi-experienced and semi-empirical methodology on the basis of considering the contributions of concrete strength, effective span to height ratio, section size, shear span–depth ratio and horizontal distributing reinforcement ratio on the shear capacity of deep beams theoretical formula. The influence of vertical stirrup on the deep beam members is not considered. The code provisions state that, for the deep beam members equipped with web reinforcements and longitudinal reinforcements, under concentrated load, the shear capacity of deep beams shall meet the following requirements:

(1) $$V\leq \frac{1.75}{\lambda +1}f_{t}bh_{0}+\frac{\left(5-l_{0}/h\right)}{6}f_{\mathrm{yh}}\frac{A_{\mathrm{sh}}}{s_{v}}h_{0}$$

To calculate the shear span–depth ratio, when *l*_0_/*h* ≤ 2,take λ = 0.25, *f*_t_ is design value for concrete axial tensile strength, *f*_yh_ is yield strength of horizontal distributing reinforcement, *A*_sh_ is cross section area; *s*_v_ is spacing of horizontal reinforcements; *l*_0_/*h* is effective span to height ratio, when *l*_0_/*h* < 2, take *l*_0_/*h* = 2.

### 4.2. American ACI318-14 Code

According to the design method of strut-and-tie model recommended in Appendix A of the American ACI318-14 Code, the shear capacity of deep beam members can be calculated by formula (2):

(2) $$F_{\mathrm{ns}}=\frac{V_{n}}{\sin\theta }$$

(3) $$F_{\mathrm{ns}}=f_{\mathrm{ce}}A_{\mathrm{ce}}$$

where *V*_n_ is the shear capacity of the deep beam. *θ* is the minimum angle between the concrete strut and the steel tie connected to it, which shall not be less than 25° as specified in the specification. *F*_ns_ is the nominal shear capacity of the strut, *f*_ce_ is the nominal compressive strength of concrete, and takes the smaller value of the effective compressive strength of the strut concrete and the effective compressive strength of concrete in the joint area. Effective compressive strength of strut concrete: *f*_ce_ = 0.85 *β*_s_*f’*_c_, effective compressive strength of concrete in joint area: *f*_ce_ = 0.85 *β*_n_*f’*_c_, *β*_s_ is the strength reduction factor after cracking of the concrete. There are three cases for the value of *β*_s_: (1) 1.0 when the compression member is of equal section; (2) 0.75 when the bottle-shaped compression rod meets the minimum reinforcement ratio requirement, and 0.6 if it does not meet the minimum reinforcement ratio requirement; (3) In other cases, 0.6λ. *β*_n_ is the reduction coefficient of the concrete strength at the joint area. There are three cases for the value of *β*_n_: (1) 1.0 when all compression is applied; (2) 0.8 for the anchoring-tie rod area; (3) 0.6 for the anchoring area of multiple tie rods. *f’*_c_ is the design value of compressive strength of concrete.

(4) $$A_{cs}=b_{w}w_{s}$$

where *b*_w_ is the section width of the specimen; *w*_s_ is the width of concrete diagonal strut, which can be calculated by Equation (5) based on Figure 10:

(5) $$w_{s}=w_{t}\cos\theta +l_{b}\sin\theta$$

(6) $$\tan\theta =\frac{h-(w_{t}+w_{t}^{\prime })/2}{a}\geq 0.488$$

where *w*_t_ and *w*’_t_ are the heights of different strut joints. *w*’_t_ = *w*_t_. In addition, according to the ACI318-14 specification, the arrangement of web reinforcement shall meet the requirements of minimum reinforcement ratio, that is:

(7) $$\sum \frac{A_{si}}{bs_{i}}\sin\theta \geq 0.003$$

where *A_si_* is the cross-sectional area of the *i* layer of web tendons; *s_i_* is the distance between the *i* layer of web reinforcements; and *θ_i_* is the angle between the *i* layer of web reinforcements and the axial direction of the concrete strut.

### 4.3. European EC Code

The calculation process of the strut-and-tie model selected in the European code is basically the same as that of the ACI318-14, only the selection of the reduction coefficient of strut and tie area is different. The relationship between compressive strength and main tensile stress is given in the code: (1) for concrete columns with or without transverse compressive stress *σ_R_*_d,max_ = *f*_cd_. (2) Transverse tensile stress and allowed to crack *σ_R_*_d,max_ = 0.6*νf*_cd_, ν = 1 − *f*_ck_/250. Where *f*_cd_ and *f*_ck_ are the values of compressive strength of concrete. It is also stipulated that the value range *θ* of deep beams should be between 45° and 75°.

### 4.4. Canadian CSAA23.3-04 Code

The calculation process of the strut-and-tie model used in the CSAA23.3-04 is basically the same as that in the ACI318-14, with only a modification of *f*_ce_. The CSAA23.3-04 states:

(8) $$f_{ce}=\frac{f_{c}^{\prime }}{0.8+170\varepsilon_{1}}\leq 0.85f_{c}^{\prime }$$

(9) $$\varepsilon_{1}=\varepsilon_{s}+(\varepsilon_{s}+0.002)\cot^{2}\theta_{s}$$

where *ε*_s_ is the tensile strain in the longitudinal tie. The other letters have the same meanings as the ACI318-14, and the range of *ε*_s_ is specified as 0.0012–0.0038.

### 4.5. Comparative Analysis of Test Results

The above-mentioned specifications are used to calculate the completed eight deep beam test specimens. Table 5 shows the performance of the four codes of practice in predicting the shear capacity with the experiment results of eight specimens. It can be seen from Table 5 the average values of the ratio of the test values to the deep beam shear capacity calculated using GB20010-2010, ACI318-14, CSSA23.3-04, and EC2 are 1.421, 1.433, 1.838, and 1.676, and the deviations are 0.027, 0.025, 0.041, and 0.032. All experimental values are lower than the theoretically obtained values. The shear capacity design equation of deep beams in GB50010-2010 neglect the effect of longitudinal reinforcement and vertical stirrup, and the shear span–depth ratio is set to be 0.25. The shear capacity derived by GB50010-2010 and ACI318-14are close to the experiment results, and the data is less discrete. The calculation results of EC2 and CSSA23.3-04 are relatively conservative, and the data is less discrete too.

## 5. Conclusions Remarks

The purpose of the current study is to obtain detailed information on shear capacity in reinforced concrete deep beams with small shear span–depth ratio by conducting the experiment of eight simply supported beam specimens. The experiment results will also allow an evaluation of the current code provisions and help identify their limitations. Based on the experimental results, the following conclusions can be derived:

(1) The shear span–depth ratio is the most important parameter that controls behavior and shear capacity of high-strength reinforced concrete deep beams.
(2) The longitudinal reinforcement ratio has no effect on the values of normal section cracking load and diagonal section cracking load of high-strength reinforced concrete deep beams, but it has a greater impact on the ultimate load. Obviously, the variation of the longitudinal reinforcement ratio applied on a beam, there will be no change of the beam’s stiffness and, therefore, the deflection will remain the same.
(3) The vertical stirrup ratio has almost no effect on the normal section cracking load, diagonal section cracking load, and ultimate load of high-strength reinforced concrete deep beams. This is consistent with the view that the vertical stirrup is not considered in the calculation formula of the shear capacity of deep beams in the GB50010-2010 and the strut-and-tie model.
(4) The presence of vertical shear reinforcement can control the crack propagation/opening and improve the ductile behavior of deep beams. But this effect is very limited as further increase in shear reinforcement does not enhance the shear capacity as failure is dominated by crushing of the concrete.
(5) In most of the specimens, the load was supported by compression strut linking with loading point and bearing point at failure. It was destroyed after the formation of diagonal cracks parallel with the strut showing brittle fracture when the diagonal compression of strut governed the failure.
(6) In the experimental results of this article, all horizontal distributing reinforcements yielded. This confirms that the horizontal distributing reinforcement is fully employed, which is consistent with the points of GB50010-2010 that the horizontal distributing reinforcement ratio is considered in the formula for calculating the shear capacity of deep beams.
(7) The calculation of shear capacity of high-strength reinforced concrete deep beams implemented by EC2 code and CSSA is mostly over-conservative, and the shear equations provided by GB20010-2010 and ACI318-14 can predict results with a reasonable degree of accuracy. The results will allow an evaluation of the current code provisions and help identify their limitations.

## Figures and Tables

**Table 1:** Mechanical properties of high-strength concrete.

*f*_cu_/MPa	*f*_c_/MPa	*f*_t_/MPa	*E*_c_/GPa
59.8	42.9	3.75	34.6

**Table 2:** Mechanical properties of steel bars.

Reinforcement	*d*/mm	*f*_y_/MPa	*f*_u_/MPa	*E*_s_/GPa
HTRB600	16	670	865	198.5
HTRB600	20	653.7	823.3	196.6
HTRB600	22	630	800	195.8
HRB400E	8	456.8	647.7	205.3

**Table 3:** Test specimen design parameters.

Component Number	*l* × *b* × *l* /mm	Shear Span–Depth Ratio *λ*	Span Height Ratio *l*_0_/*h*	Longitudinal Reinforcement Ratio *ρ*_s_ (%)	Horizontal Reinforcement Ratio *ρ*_sh_ (%)	Vertical Stirrup Ratio *ρ*_sv_ (%)
MDB1	1600 × 200 × 600	0.3	2	1.05	0.33	0.33
MDB2	1600 × 200 × 600	0.6	2	1.05	0.33	0.33
MDB3	1600 × 200 × 600	0.9	2	1.05	0.33	0.33
MDB4	1600 × 200 × 600	0.6	2	0.67	0.33	0.33
MDB5	1600 × 200 × 600	0.6	2	1.27	0.33	0.33
MDB6	1600 × 200 × 600	0.6	2	1.05	0.33	0.50
MDB7	1600 × 200 × 600	0.6	2	1.05	0.33	0.25
MDB8	1600 × 200 × 600	0.6	2	1.05	0.33	0

**Table 4:** Main test results of MDB series deep beam specimens.

Specimen	$V_{\mathrm{cr}}^{N}/\mathrm{kN}$	$V_{\mathrm{cr}}^{D}/\mathrm{kN}$	$V_{u}/\mathrm{kN}$	$V_{\mathrm{cr}}^{N}$ $/V_{u}$	$V_{\mathrm{cr}}^{D}$ $/V_{u}$	δ/mm	Failure Model	Failure Form
MDB1	359	370	1100.0	32.64%	33.64%	3.15	Diagonal-compression	Crushing-strut
MDB2	138	188	903.5	15.27%	20.81%	4.00	Diagonal-compression	Crushing-strut
MDB3	99	149	785.0	12.61%	18.98%	5.99	Diagonal-compression	Crushing-strut
MDB4	139	299	750.0	18.53%	39.87%	3.12	Diagonal-compression	Crushing-strut
MDB5	149	184	993.7	14.99%	18.52%	3.82	Diagonal-compression	Crushing-strut
MDB6	199	300	933.0	21.33%	32.15%	3.29	Diagonal-compression	Crushing-strut
MDB7	145	199	899.0	19.33%	22.14%	3.80	Diagonal-compression	Crushing-strut
MDB8	165	170	893.5	18.47%	19.03%	2.98	Diagonal-compression	Diagonal-splitting

Note: The loads in the table are only for one side of the deep beam during the test. $V_{\mathrm{cr}}^{N}$ is the normal section cracking load; $V_{\mathrm{cr}}^{D}$ is the diagonal section cracking load; $V_{u}$ is the ultimate load; δ is the mid-span deflection.

**Table 5:** Comparison of prediction values and experimental values.

Specimen	Test Value	Predictions V_n_(kN)				V_test_/V_n_			
		China	ACI	EC2	CSA	China	ACI	EC2	CSA
MDB1	1100	638.54	620.09	483.46	659.06	1.723	1.774	2.275	1.669
MDB2	903.5	638.54	644.37	502.40	555.24	1.415	1.402	1.798	1.627
MDB3	785	638.54	583.68	455.08	382.33	1.229	1.345	1.725	2.053
MDB4	750	640.94	634.49	494.69	548.45	1.170	1.182	1.516	1.367
MDB5	993.7	637.34	649.32	506.26	558.61	1.559	1.530	1.963	1.779
MDB6	933	638.54	644.37	502.40	555.24	1.461	1.448	1.857	1.680
MDB7	899	638.54	644.37	502.40	555.24	1.408	1.395	1.789	1.619
MDB8	893.5	638.54	644.37	502.40	555.24	1.399	1.387	1.778	1.609
Mean						1.421	1.433	1.838	1.676
variance						0.027	0.025	0.041	0.032

## References

[1] (2010). Design Code for Concrete Structures.

[2] Deep Beam Group (1987). Experimental study on reinforced concrete deep beam. J. Build. Struct..

[3] Qian G.L. (1980). Experimental study on reinforced concrete simply supported deep beams. J. Wuhan Inst. Water Resour. Electr. Power.

[4] Liu L.X., Gong S.X. (1991). Ultimate analysis of shear capacity of reinforced concrete simply supported deep beams with web openings. J. Build. Struct..

[5] Liu L.X. (1995). An unified calculation method for shear capacity of reinforced concrete deep beam short beam and shallow beams. J. Build. Struct..

[6] Marti P. (1985). Basic tools of reinforced concrete beam design. ACI Struct. J..

[7] Cook W.D., Mitchell D. (1988). Studies of disturbed regions near discontinuities in reinforced concrete members. ACI Struct. J..

[8] Schlaich J., Schafer K., Jennewein M. (1987). Toward a consistant design of structural concrete. Prestress. Concr. Inst. J..

[9] Vecchio F.J., Collins M.P. (1986). The modified compression field theory for reinforced concrete elements subjected to shear. ACI J. Proc..

[10] Vecchio F.J., Collins M.P. (1993). Compression response of cracked reinforced concrete. J. Struct. Eng. ASCE.

[11] Hwang S.J., Lu W.Y., Lee H.J. (2000). Shear strength prediction for deep beams. ACI Struct. J..

[12] Hwang S.J., Lee H.J. (2002). Strength predication for discontinuity regions by soften strut-and-tie model. J. Struct. Eng. ASCE.

[13] Bažant Z.P., Planas J. (1998). Fracture and Size Effect in Concrete and Other Quasibrittle Materials.

[14] Yang K.H., Ashour A.F. (2010). Strut-and-tie model based on crack band theory for deep beams. J. Struct. Eng..

[15] (2014). ACI Committee 318: Building Code Requirement for Structure Concrete (ACI 318–14) and Commentary.

[16] (2004). The European Standard EN 1992–1–1:2004, Eurocode 2, Design of Concrete Structures.

[17] (2004). Design of Concrete Structures.

[18] Tang C.Y., Tan K.H. (2004). Interactive mechanical model for shear strength of deep beams. J. Struct. Eng. ASCE.

[19] Tan K.H., Cheng G.H. (2006). Size effect on shear strength of deep beams: Investigating with strut-and-tie model. J. Struct. Eng. ASCE.

[20] Liu L.Q., Wang J.J., Han J.Y., Zhang G.X., Wu X.L. (2013). Experimental study on static performance of reinforced concrete simply-supported deep beams and analysis of compression rod model. J. Build. Struct..

[21] Qiu Y.K., Liu X., Lin Y. (2012). Experimental study on simply supported reinforced concrete deep beams of strut-and-tie models. Build. Struct..

[22] Lu W.Y., Lin I.J., Yu H.W. (2013). Shear strength of reinforced concrete deep beams. ACI Struct. J..

[23] Tan K.H., Cheng G.H., Zhang N. (2007). Experiment to mitigate size effect on deep beams. Mag. Concr. Res..

[24] De Dios Garay J., Lubell A.S. Behavior of Concrete deep beams with high strength reinforcement. Proceedings of the American Society of Civil Engineers, Structures Congress 2008.

[25] Yang K.H., Chung H.S., Lee E.T. (2003). Shear characteristics of high strength concrete deep beams without shear reinforcements. Eng. Struct..

[26] Chen H., Yi W.J., Ma Z.J., Hwang H.J. (2019). Shear strength of reinforced concrete simple and continuous deep beams. ACI Struct. J..

[27] Sanabria Díaz R.A., Sarmiento Nova S.J., Teixeira da Silva M.C.A., Mouta Trautwein L., de Almeida L.C. (2020). Reliability analysis of shear strength of reinforced concrete deep beams using NLFEA. Eng. Struct..

[28] Placidi L., Barchiesi E. (2018). Energy approach to brittle fracture in strain-gradient modelling. Proc. R. Soc. A Math. Phys. Eng. Sci..

[29] Placidi L., Barchiesi E., Misra A. (2018). A strain gradient variational approach to damage: A comparison with damage gradient models and numerical results. Math. Mech. Complex Syst..

[30] Nguyen T.H., Niiranen J. (2020). A second strain gradient damage model with a numerical implementation for quasi-brittle materials with micro-architectures. Math. Mech. Solids.

[31] Tang X.R., Sun H.M. (2010). Experimental research and design suggestion on static behavior of spatial steel frame concrete simply supported deep beam. J. Build. Struct..

[32] Wu T., Wei H., Liu X. (2020). Shear behavior of large-scale deep beams with lightweight-aggregate concrete. ACI Struct. J..

[33] Gao D.Y., Zhao J., Zhu H.T., Zhang Q.M. (2002). Calculation method for crack resistance of steel fiber concrete deep beams with reinforcement. J. Hydraul. Eng..

[34] Zhao J., Zhu H.T., Gao D.Y. (2003). The calculating method of shear capacity steel fiber reinforced concrete deep beams. Henan Sci..

[35] Ma K.Z., Qi T., Liu H.J., Wang H.B. (2018). Shear behavior of hybrid fiber reinforced concrete deep beams. Materials.

[36] Ferdous W., Almutairi A.D., Huang Y., Bai Y. (2018). Short-term flexural behaviour of concrete filled pultruded GFRP cellular and tubular sections with pin-eye connections for modular retaining wall construction. Compos. Struct..

[37] Ferdous W., Bai Y., Almutairi A.D., Satasivam S., Jeske J. (2018). Modular assembly of water-retaining walls using GFRP hollow profiles: Components and connection performance. Compos. Struct..

[38] Zhao G.F. (1988). Advanced Reinforced Concrete Structure.

[39] Park R., Paulay T. (1985). Reinforced Concrete Structure.

[40] Ferdous W., Manalo A., Aravinthan T. (2017). Effect of beam orientation on the static behavior of phenolic core sandwich composites with different shear span-to-depth ratios. Compos. Struct..

[41] Yang K.H., Chung H.S., Ashour A.F. (2007). Influence of shear reinforcement on reinforced concrete continuous deep beams. ACI Struct. J..

